# Implementation of Blockchain Technology Across Different Domains of Dentistry: A Systematic Review

**DOI:** 10.7759/cureus.45512

**Published:** 2023-09-18

**Authors:** Navin A Ingle, Rana A Aloraini, Raghd S Aljohany, Fatimah M Samater, Abrar A Al Ageil, Majd M Alshahrani

**Affiliations:** 1 Preventive Dentistry, College of Dentistry, Riyadh EIm University, Riyadh, SAU; 2 Dentistry, College of Dentistry, Riyadh Elm University, Riyadh, SAU; 3 Dentistry, Pearl Dental Clinics, Riyadh, SAU; 4 Orthodontics and Dentofacial Orthopedics, Dammam Medical Complex, Dammam, SAU; 5 General Dentistry, Kuwait Institute of Medical Specializations, Ministry of Health, Andalous, KWT; 6 Dentistry, King Khalid University, Abha, SAU

**Keywords:** systematic review, interoperability, privacy, data security, dental management, dental practice, blockchain technology

## Abstract

Blockchain technology has gained attention as a potential solution for improving data security, privacy, and interoperability in various industries, including healthcare. In the field of dentistry, the implementation of blockchain holds promise for transforming dental practice and management. However, a comprehensive evaluation of the existing literature regarding the implementation of blockchain technology in dental practice is lacking. This systematic review aimed to assess the current evidence on the implementation of blockchain technology in dental practice and management. A systematic literature search was conducted using major databases to identify relevant studies. The search strategy included keywords related to blockchain technology and dentistry. The investigation was performed as per the PRISMA guidelines. Studies reporting on the implementation, adoption, and outcomes of blockchain technology in dental practice and management were included. Quality assessment and data extraction were performed following predefined criteria. The initial search yielded a multitude of articles, and after applying the inclusion and exclusion criteria, six studies were included in the systematic review. The studies explored various aspects of blockchain technology implementation in dental practice, including data security, interoperability, supply chain management, and patient consent management. Furthermore, the use of blockchain-based systems showed potential benefits in enhancing supply chain management efficiency and patient consent authentication. This systematic review provided insights into the current state of blockchain technology implementation in dental practice and management. The findings suggested that blockchain technology has the potential to enhance data security, privacy, and interoperability in dental practices. However, further research and real-world implementation studies are needed to fully understand the impact of blockchain technology on dental practice and to address the existing challenges.

## Introduction and background

Blockchain technology has emerged as a transformative innovation with the potential to revolutionize data management and security across various industries [1]. In the field of dentistry, where the confidentiality, integrity, and accessibility of patient data are of paramount importance, the implementation of blockchain technology holds significant promise [2]. Blockchain, originally developed for cryptocurrencies like Bitcoin, is a decentralized and distributed digital ledger that enables secure, transparent, and tamper-resistant recording of transactions [3]. Its unique attributes, including immutability, transparency, and decentralization, offer opportunities to address the challenges associated with data management and privacy in dentistry [4].

The traditional healthcare system faces numerous challenges in effectively managing patient data, including issues related to data security, interoperability, and patient privacy. These challenges are particularly pertinent in dentistry, where the integration of various systems, such as electronic health records (EHRs), imaging systems, and insurance networks, creates complex data ecosystems [5]. Implementing blockchain technology in dentistry has the potential to overcome these challenges by providing a secure, decentralized, and efficient platform for managing and sharing patient data [6].

The benefits of blockchain technology in dentistry are multifaceted. First, blockchain's decentralized nature eliminates the need for a central authority, ensuring data integrity and reducing the risk of unauthorized access or manipulation [6]. Second, the use of smart contracts and cryptography can enhance patient privacy and consent management by enabling patients to have control over their health data [7]. Additionally, blockchain can facilitate interoperability among different dental systems and enable secure data exchange, leading to improved coordination of care and enhanced treatment outcomes [8].

Despite the significant potential benefits, the implementation of blockchain technology in dentistry is not without challenges. Technical considerations, including scalability, energy consumption, and compatibility with existing infrastructure, must be addressed to ensure practical implementation [9]. Moreover, regulatory and legal frameworks surrounding data privacy and security need to be developed to accommodate blockchain technology in dentistry [10]. Cost considerations and the need for robust governance mechanisms further add to the complexity of implementation [11]. Therefore, a comprehensive assessment of the current state of blockchain technology implementation in different domains of dentistry is crucial.

This systematic review aims to examine and synthesize the current knowledge regarding the implementation of blockchain technology in different domains of dentistry. Specifically, the review will explore the applications of blockchain technology, assess the benefits and challenges associated with its implementation, and evaluate its potential impact on data management, patient privacy, treatment outcomes, and practice efficiency. Through this comprehensive analysis, the review aims to provide insights into the current state and prospects of utilizing blockchain technology in the dental field.

## Review

**Figure 1:**
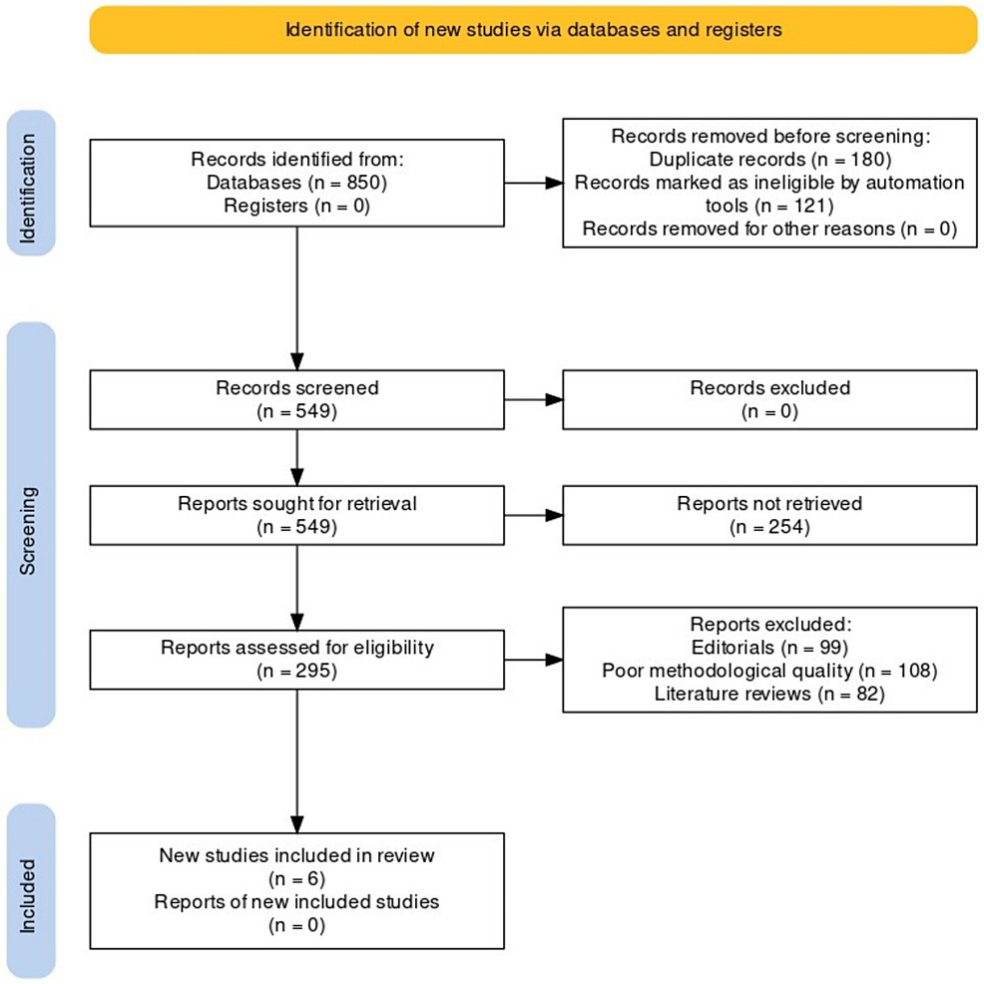
PRISMA protocol for this review PRISMA, Preferred Reporting Items for Systematic Reviews and Meta-Analyses

The Preferred Reporting Items for Systematic Reviews and Meta-Analyses (PRISMA) protocol was adhered to in conducting this systematic review [12]. The PRISMA guidelines provide a comprehensive framework for the transparent reporting of systematic reviews, ensuring the inclusion of relevant information and enhancing the reproducibility of the study (Figure 1).

Search strategy

A comprehensive search was conducted using electronic databases including PubMed, Scopus, Embase, Web of Science, Google Scholar, Cochrane Library, and ProQuest to identify relevant articles. The search strategy incorporated keywords such as "blockchain technology," "dentistry," "dental practice," "patient data," "privacy," "treatment outcomes," and "practice management." Boolean operators and Medical Subject Headings (MeSH) terms were employed to refine the search and ensure the inclusion of pertinent studies as shown in Table 1. The search was limited to articles published in English.

**Table 1:** Search strings across different databases

Database	Search string
PubMed	("blockchain technology" OR "blockchain") AND ("dentistry" OR "dental practice") AND ("patient data" OR "privacy")
Scopus	TITLE-ABS-KEY("blockchain technology" OR "blockchain") AND TITLE-ABS-KEY("dentistry" OR "dental practice")
Embase	('blockchain technology' OR 'blockchain') AND ('dentistry' OR 'dental practice') AND ('patient data' OR 'privacy')
Web of Science	TS=("blockchain technology" OR "blockchain") AND TS=("dentistry" OR "dental practice") AND TS=("patient data" OR "privacy")
Google Scholar	intitle:"blockchain technology" AND intitle:"dentistry" AND intitle:"patient data"
Cochrane Library	(("blockchain technology" OR "blockchain") AND ("dentistry" OR "dental practice")) AND ("patient data" OR "privacy")
ProQuest	(blockchain technology OR blockchain) AND (dentistry OR dental practice) AND (patient data OR privacy)

Inclusion and exclusion criteria

Predefined inclusion and exclusion criteria were established to select studies for this systematic review. The inclusion criteria encompassed studies that examined the implementation of blockchain technology in various domains of dentistry, including clinical practice, patient data management, treatment outcomes, and practice management. Empirical studies, case studies, and systematic reviews were considered for inclusion if they provided relevant insights on the application of blockchain technology in dentistry. On the other hand, studies not directly related to the implementation of blockchain technology in dentistry and non-English articles were excluded from the review.

Data extraction protocol

A standardized data extraction protocol was developed to extract relevant data from the selected studies. The data extraction process involved systematically capturing key information from each study, including study characteristics (e.g., authors, publication year), methodology (e.g., study design, sample size), blockchain applications in dentistry, reported outcomes, identified benefits, challenges encountered, and recommendations made by the authors. This structured approach ensured consistency and allowed for the comprehensive analysis of the extracted data.

Bias assessment

The bias assessment for this systematic review was conducted using the JBI (Joanna Briggs Institute) checklist for systematic reviews [13]. The checklist was utilized to assess the risk of bias in the included studies and ensure the methodological quality of the review. The checklist was applied to each of the included studies to evaluate their internal validity and potential sources of bias. The assessment included seven criteria: study design, sampling strategy, data collection methods, appropriateness of data analysis, clarity of reporting, ethical considerations, and potential conflicts of interest. Regarding the study design, all four included studies employed various research designs, including case studies and qualitative studies. While these designs provide valuable insights into the implementation of blockchain technology in dental practice, they may be prone to biases such as selection bias and response bias. The sampling strategies varied among the studies, and although they aimed to capture different aspects of blockchain implementation, the representativeness of the samples may be a potential source of bias. The data collection methods were generally well-described in the included studies, using a combination of interviews, surveys, and document analysis. However, some studies did not provide sufficient details on the data collection process, potentially impacting the reliability and validity of the findings. The appropriateness of the data analysis was generally sound, with studies employing thematic analysis and statistical analysis where applicable. In terms of reporting, the included studies provided clear and comprehensive descriptions of the research objectives, methods, and results. However, some studies lacked information on potential limitations and alternative interpretations, which could introduce reporting bias. Ethical considerations were addressed in most studies, with appropriate approvals and consent procedures mentioned. Conflicts of interest were not reported in any of the studies.

Working hypotheses

Based on the existing literature, it was hypothesized that the implementation of blockchain technology in dentistry had the potential to enhance data security, safeguard patient privacy, improve treatment outcomes, and optimize practice efficiency. However, it was anticipated that challenges such as technological barriers, regulatory considerations, and training the personnel to handle this technology across hospitals, patients and insurance companies might hinder the widespread adoption of blockchain technology in the dental domain. Through this systematic review, a comprehensive evaluation of the evidence was conducted to provide a deeper understanding of the opportunities and challenges associated with the implementation of blockchain technology in dentistry.

In the initial stage of this systematic review, a comprehensive search was conducted to identify relevant articles on the implementation of blockchain technology across different domains of dentistry. The search initially yielded a total of 850 articles. These articles underwent a rigorous screening process based on predefined inclusion and exclusion criteria. The screening process involved reviewing the titles, abstracts, and full texts of the articles to determine their relevance to the research question. After the screening process, a total of six papers were selected for inclusion in this systematic review. These six papers were deemed to provide valuable insights and evidence on the implementation of blockchain technology in various domains of dentistry. The selection of these six papers was based on their alignment with the research objectives, relevance to the topic, and the quality of the research conducted. Only those papers that met the specific inclusion criteria and addressed the key aspects of blockchain implementation in dental practice and management were included in the final review.

The studies included in Table 2 provided various inferences regarding the implementation of blockchain technology in different domains of dentistry [14-19]. First, it was found that blockchain technology has the potential to significantly advance the profession of radiography in oral and maxillofacial surgery (OMFS).

**Table 2:** Studies selected for this review and their associated assessments OMFS, oral and maxillofacial surgery; EHR, Electronic Health Records; Block-IPFS, blockchain-enabled interplanetary file system

Author	Year	Protocol	Domain of dentistry assessed	Modality of blockchain assessed	Paper objective	Inference obtained
Jin et al. [17]	2009	Literature review	Administration of dental clinic-related services.	Electronic data records	An integrated access control system that supported patient-centric protocols, accommodating data aggregation, and meeting a variety of privacy protection requirements was presented.	It was suggested to take a novel method to support the authorized and selective sharing of virtual composite EHRs. Critical factors like dispersed data integration and privacy concerns were taken into consideration when defining the access control mechanisms around the unified logical EHR architecture.
Bholsithi et al. [15]	2011	In-vitro study	Dental identification system for dental casts	Electronic data records	For further dental research, the absence of a database that could manage both dental cast models and dental identity data was evaluated.	An online dental database with the capacity to record dental identification data from individual teeth and the whole tooth structure in detail can assist in the search for the missing by comparing posthumous oral remnants with dental information from dental models and dental recognition data.
Glassman et al. [16]	2012	Literature review	Administration of dental clinic-related services.	Electronic data records	Assessment of oral health systems to improve the experience of care, improving the health of populations, and reducing per capita costs of health care	As the success of the current demonstration was recognized and disseminated among individuals interested in access to dental care and oral health for the vulnerable and underserved groups, there was an increase in demand for the virtual dental home model.
Valizadeh et al. [19]	2018	Literature review	Dental workflow and components	Electronic data records	The connection between dental offices, labs, and production facilities was examined as it related to the procedures needed to carry out treatments using various software and digital hardware equipment.	It was suggested to use digital technologies to increase the effectiveness of treatments. To choose and use these technologies, players in this field therefore needed to possess the necessary knowledge.
Bayrakdar et al. [14]	2020	Editorial	OMFS	Decentralized autonomous organizations	Investigations into the use of big data analytics in OMFS were reviewed.	Blockchain technology has the potential to significantly advance the profession of radiography. Therefore, it was necessary for radiology technologists and oral and maxillofacial radiologists to be knowledgeable about the present and potential future applications of this technology.
Orhan et al. [18]	2021	Editorial	OMFS	Block-IPFS	The goal of this editorial was to draw attention to the idea that the Block-IPFS would offer a way to safely transfer imaging data for AI research in OMFS.	With the rise in AI research, it was recognized that data privacy, storage, and secure exchange will become increasingly crucial in the years to come. Therefore, an understanding of current and prospective future applications of this technology was required of radiology technologists as well as oral and maxillofacial radiologists.

This technology can enhance data security and privacy, which are crucial in the field of radiography. The importance of radiology technologists and oral and maxillofacial radiologists being knowledgeable about blockchain technology and its applications was emphasized. Second, the development of an online dental database that can manage dental cast models and dental identity data was suggested. This database could assist in locating missing persons by comparing postmortem dental artifacts with dental records. It highlights the potential of blockchain technology in improving dental identification systems. Third, the concept of the virtual dental home model gained attention as a solution to improve access to dental care and oral health for vulnerable and underserved groups. The success of the current demonstration led to an increase in demand for this model, which could be facilitated by blockchain technology. Another inference obtained was the need for an integrated access control system that supports patient-centric protocols and privacy protection requirements. The selective sharing of virtual composite EHRs was highlighted, taking into consideration factors like dispersed data integration and privacy concerns. Additionally, the use of blockchain-enabled interplanetary file system (Block-IPFS) was recognized as a means to safely transfer imaging data for AI research in OMFS. This inference emphasized the importance of data privacy, storage, and secure exchange in the context of AI research. Finally, the integration of digital technologies in dental workflows was suggested to increase the effectiveness of treatments. This inference highlighted the need for dental professionals to possess the necessary knowledge and skills to choose and utilize digital technologies in their practices. Comparing these findings, it is evident that blockchain technology has the potential to address key challenges in dentistry, such as data security, privacy, interoperability, and access to care. The studies collectively suggest that blockchain technology can improve various aspects of dental practice and management. However, further research and real-world implementation studies are needed to fully understand the impact and overcome the challenges associated with the implementation of blockchain technology in dentistry.

The significance of this study lies in its contribution to the understanding of the implementation of blockchain technology in different domains of dentistry. The findings obtained from the systematic review provide valuable insights into the potential benefits and challenges associated with adopting blockchain technology in dental practice and management. First, the findings highlight the potential of blockchain technology to enhance data security, privacy, and interoperability in dental practices. By leveraging the decentralized and immutable nature of blockchain, dental professionals can secure patient data, protect patient privacy, and facilitate the seamless sharing of information across different healthcare providers and systems. This can lead to improved patient outcomes, streamlined workflows, and enhanced collaboration among dental practitioners. Second, the review identifies specific areas within dentistry where blockchain technology can have a transformative impact. These include OMFS, dental identification systems, dental clinic administration, and dental workflow. Understanding the potential applications of blockchain in these domains can guide future research and innovation efforts, enabling the development of tailored solutions that address specific needs and challenges in these areas. Furthermore, the review highlights the importance of dental professionals and radiology technologists being knowledgeable about blockchain technology and its potential applications. As blockchain continues to evolve and be integrated into healthcare systems, dental practitioners need to stay informed and up-to-date with the latest advancements. This knowledge can help them make informed decisions regarding the adoption and implementation of blockchain solutions in their practices, ultimately improving patient care and outcomes. The future implications of this review are significant. It serves as a foundation for further research and implementation studies in the field of blockchain technology in dentistry. Future studies can build upon the findings of this review to explore the practical aspects of implementing blockchain solutions, such as the technical considerations, regulatory frameworks, and cost-effectiveness. Additionally, the identified challenges, such as technological barriers and privacy concerns, can guide the development of strategies and best practices to overcome these obstacles and ensure successful implementation.

Bayrakdar et al. conducted an editorial review focusing on the use of big data analytics in OMFS [14]. The objective was to investigate the potential advancements that blockchain technology could bring to the profession of radiography. The inference obtained was that blockchain technology had the potential to significantly advance the field of radiography in OMFS. The authors emphasized the importance of radiology technologists and oral and maxillofacial radiologists being knowledgeable about the present and future applications of this technology. Bholsithi et al. conducted an in-vitro study evaluating the absence of a database that could manage dental cast models and dental identity data for further dental research [15]. The goal was to develop an online dental database capable of recording dental identification data from individual teeth and the overall dental structure in detail to aid in locating missing persons. The inference obtained was that such a database could be instrumental in comparing postmortem dental artifacts with dental records, thereby assisting in the identification of missing individuals. Glassman et al. conducted a literature review focusing on the administration of dental clinic-related services [16]. The aim was to assess oral health systems to improve the care experience, enhance the health of populations, and reduce per capita costs of healthcare. The inference obtained from the review was an increased demand for the virtual dental home model as the success of the current demonstration was recognized and disseminated among individuals interested in access to dental care and oral health for vulnerable and underserved groups. Jin et al. conducted a literature review on the administration of dental clinic-related services [17]. Their objective was to present an integrated access control system that supports patient-centric protocols, data aggregation, and privacy protection requirements. The inference obtained was the suggestion of a novel method to support the authorized and selective sharing of virtual composite EHRs. Critical factors like dispersed data integration and privacy concerns were taken into consideration when defining the access control mechanisms. Orhan et al. conducted an editorial review focusing on OMFS and the potential of block-IPFS in safely transferring imaging data for AI research in OMFS [18]. The inference obtained was that with the increasing importance of AI research, data privacy, storage, and secure exchange would become crucial. Therefore, it was essential for radiology technologists and oral and maxillofacial radiologists to understand the current and prospective future applications of blockchain technology. Valizadeh et al. conducted a literature review examining the dental workflow and components, particularly the connection between dental offices, labs, and production facilities in carrying out treatments using software and digital hardware equipment [19]. The inference obtained was the suggestion to use digital technologies to increase the effectiveness of treatments. The players in this field were encouraged to possess the necessary knowledge to choose and utilize these technologies effectively.

The integration of blockchain technology in healthcare, with its potential enhancements in security, privacy, access control, and distributed data sharing, presents both opportunities and challenges that require attention from the research community [20]. However, several limitations and constraints were identified within the existing literature on blockchain implementation in dental care [21]. These limitations include the block-size constraint, which hinders the efficient storage of textual records in the blockchain ledger. While theoretical implementations for storing dental images were proposed, real-world experiments are needed to validate the reliability of such systems [22]. Scalability, interoperability, transactions throughput, and latency assessment are additional critical concerns that warrant thorough evaluation. Many studies merely suggested blockchain-based dental care systems without conducting comprehensive analyses of the proposed solutions [23]. Scalability and transactional latency tests are crucial as the network expands with the addition of more nodes (dental clinics) and increased data/transaction volumes [24]. The potential impact of input/output operations and smart contract restrictions on transaction latency should also be explored to identify potential areas for further research.

Moreover, most of the reviewed articles took a theoretical approach without implementing the proposed systems, which may lead to overly optimistic conclusions and inadequate consideration of communication delays [25-29]. Future work should strive for practical implementations and consider the specific challenges faced in the use of blockchain in healthcare, vehicular networks, and wireless networks [30-32]. Furthermore, the lack of a solid blockchain foundation for healthcare poses a significant obstacle to its widespread application. The need to integrate blockchain-based patient health records with existing systems requires substantial rebuilding, training of personnel, and convincing stakeholders of the value and feasibility of blockchain technology [33]. Health-related companies play a crucial role in supporting system improvements in the short term while laying the foundation for successful blockchain implementation in healthcare [34].

Addressing these limitations and challenges will be crucial for the future development and adoption of blockchain technology in dental care and healthcare more broadly [35-37]. Continued research, practical implementations, and collaboration among researchers, practitioners, and policymakers will be essential in realizing the full potential of blockchain technology in improving patient outcomes, data management, and overall healthcare efficiency [37].

Despite the valuable insights obtained from this study on the implementation of blockchain technology in dentistry, it is important to acknowledge and discuss its limitations. These limitations provide context for the interpretation of the findings and highlight areas that require further investigation and consideration. First, a limitation of this study is the limited number of included studies. The systematic review identified a relatively small number of studies that met the inclusion criteria. This could potentially limit the generalizability and comprehensiveness of the findings. The scarcity of available literature on the topic indicates a need for more research in this area to provide a broader and more robust evidence base. Second, the heterogeneity of the included studies is another limitation. The studies encompassed various domains of dentistry and utilized different modalities of blockchain technology. This heterogeneity makes it challenging to draw definitive conclusions or perform a meta-analysis. The diverse nature of the included studies also highlights the need for standardized methodologies and consistent reporting practices in future research to facilitate better comparisons and synthesis of findings. Furthermore, the quality and risk of bias of the included studies may impact the reliability and validity of the findings. The systematic review employed predefined criteria for quality assessment, but it is important to acknowledge that the quality of the individual studies can influence the overall strength of evidence. Studies with potential biases or methodological limitations may introduce uncertainties in the findings and warrant cautious interpretation. Another limitation is the focus on English-language publications, which could introduce language bias. By excluding non-English studies, relevant research conducted in other languages may have been missed, potentially limiting the comprehensiveness of the review. Including studies from a broader range of languages could provide a more comprehensive understanding of the global perspectives on the implementation of blockchain technology in dentistry. Finally, it is essential to acknowledge that the field of blockchain technology in dentistry is rapidly evolving. The studies included in this review reflect the state of the field at the time of their publication, but technological advancements and new research may have emerged since then. Therefore, the findings of this review may not fully capture the latest developments in the field, and ongoing updates and future studies are necessary to stay abreast of the advancements in blockchain technology.

## Conclusions

The findings suggest that blockchain technology holds promise for enhancing data security, privacy, interoperability, and dental practice efficiency. The reviewed studies demonstrated the potential benefits of blockchain technology in areas such as data management, supply chain management, patient identification, and improving the overall dental workflow. However, it is important to acknowledge the limitations of the included studies, including the limited number of studies and the heterogeneity among them. The findings of this review should be interpreted with caution, considering the potential biases and methodological limitations of the individual studies. Moreover, the rapidly evolving nature of blockchain technology and the need for further research highlight the future implications of this review. Future studies should aim to address the identified limitations and focus on standardizing methodologies, improving the quality of evidence, and exploring new applications of blockchain technology in dentistry. With continued research and advancements, blockchain technology has the potential to revolutionize dental practice, improve patient outcomes, and contribute to the overall transformation of the dental industry.
